# Trajectories of Heart Activity Across Infancy to Early Childhood Differentially Predict Autism and Anxiety Symptoms in Fragile X Syndrome

**DOI:** 10.3389/fpsyt.2021.727559

**Published:** 2021-10-06

**Authors:** Abigail Hogan, Erin Hunt, Kayla Smith, Conner Black, Katherine Bangert, Jessica Klusek, Jane Roberts

**Affiliations:** ^1^Department of Psychology, University of South Carolina, Columbia, SC, United States; ^2^Department of Communication Sciences and Disorders, Arnold School of Public Health, University of South Carolina, Columbia, SC, United States

**Keywords:** autonomic nervous system, respiratory sinus arrhythmia, interbeat interval, hyperarousal, fragile X syndrome, autism spectrum disorder, anxiety

## Abstract

**Background:** Fragile X syndrome (FXS) is a monogenic disorder characterized by high rates of autism spectrum disorder (ASD) and anxiety. A longstanding “hyperarousal hypothesis” in FXS has argued that ANS dysfunction underpins many symptoms of FXS. However, the developmental onset and trajectory of ANS dysfunction, as well as the consequences of ANS dysfunction on later psychiatric symptoms, remain poorly understood in FXS. Insight into the emergence, trajectory, and consequences of ANS dysfunction across early development in FXS has critical implications for prevention, intervention, and optimal outcomes in both typical and atypical development. This longitudinal study investigated whether and when males with FXS evidence atypical ANS function from infancy through early childhood, and how trajectories of ANS function across infancy and early childhood predict ASD and anxiety symptom severity later in development.

**Methods:** Participants included 73 males with FXS and 79 age-matched typically developing (TD) males. Baseline heart activity was recorded at multiple assessments between 3 and 83 months of age, resulting in 372 observations. General arousal and parasympathetic activity were indexed via interbeat interval (IBI) and respiratory sinus arrhythmia (RSA), respectively. ASD and anxiety symptoms were assessed at 36 months of age or later in a subgroup of participants (FXS *n* = 28; TD *n* = 25).

**Results:** Males with FXS exhibited atypical patterns of developmental change in ANS function across infancy and early childhood. As a result, ANS dysfunction became progressively more discrepant across time, with the FXS group exhibiting significantly shorter IBI and lower RSA by 29 and 24 months of age, respectively. Shorter IBI at 24 months and a flatter IBI slope across development predicted elevated anxiety symptoms, but not ASD symptoms, later in childhood in both FXS and TD males. Reduced RSA at 24 months predicted elevated ASD symptoms, but not anxiety symptoms, in both groups. Developmental change in RSA across early development did not predict later anxiety or ASD symptoms.

**Conclusion:** This is the first longitudinal study to examine the “hyperarousal hypothesis” in infants and young children with FXS. Findings suggest that hyperarousal (i.e., shorter IBI, lower RSA) is evident in males with FXS by 24–29 months of age. Interestingly, unique aspects of early ANS function differentially relate to later ASD and anxiety symptoms. General arousal, indexed by shorter IBI that becomes progressively more discrepant from TD controls, predicts later anxiety symptoms. In contrast, parasympathetic-related factors, indexed by lower levels of RSA, predict ASD symptoms. These findings support the “hyperarousal hypothesis” in FXS, in that ANS dysfunction evident early in development predicts later-emerging symptoms of ASD and anxiety. This study also have important implications for the development of targeted treatments and interventions that could potentially mitigate the long-term effects of hyperarousal in FXS.

## Introduction

The autonomic nervous system (ANS) is primarily responsible for maintaining physiological balance in the human body by mobilizing the body's resources in the face of challenge or stress and facilitating physiological rest and relaxation when that challenge or stress has abated (1–4). When the sympathetic nervous system (SNS) and parasympathetic nervous system (PNS) of the ANS are balanced and functioning optimally, our bodies respond flexibly to and recover quickly from stress (5). Cardiac indices of ANS function provide unique, moment-to-moment insight into SNS and PNS influences on the heart (6, 7); specifically, interbeat interval (IBI), the time between successive heartbeats, indexes heart rate (HR) and is a reflection of general arousal, which is influenced by both the PNS and SNS (5, 6, 8). Alternatively, respiratory sinus arrhythmia (RSA), the variability in IBI that corresponds with spontaneous breathing, indexes heart rate variability (HRV) and more specifically PNS regulation of the heart via the vagal nerve (2, 4, 9–12). The ANS underlies processes related to attention as well as emotional and behavioral regulation (5, 13–15). Furthermore, at baseline and in the absence of challenge, stronger vagal regulation of the heart, reflected in higher RSA, is thought to support social engagement and has been associated with flexible response to stress, adaptive functioning, and prosocial behavior (4, 16). Thus, chronic imbalance between the two branches of the ANS has significant cascading effects on emotional, behavioral, and cognitive development as well as overall physical well-being (17, 18). Given the connection between the ANS and emotion regulation, it is unsurprising that ANS dysfunction has been linked to several psychiatric conditions characterized by emotion regulation difficulties, such as anxiety disorders and autism spectrum disorder (ASD) (9, 19).

Much of what is known about ANS function and its role in psychiatric disorders comes from studies of older TD children and adults; however, emerging evidence from longitudinal studies has provided some insight on trajectories of ANS function across early neurotypical development. For example, Bar-Haim et al. (20) examined stability and developmental change in baseline IBI and HRV across the first four years of life in TD children. Interbeat interval was shown to increase steadily from four months to four years, with the greatest increase between four and nine months. Heart rate variability also increased steadily across this age range. In a series of longitudinal studies, Patriquin et al. (21, 22) characterized baseline RSA across the first four years of life in relation to a variety of social-emotional and behavioral symptoms at age four. Results revealed that developmental trajectories of baseline RSA could be best characterized by two patterns, a “typical” pattern, in which RSA increased steadily across development, and an “atypical” pattern, in which RSA was consistently lower or plateaued over time. Of relevance to the current study, the children with atypical RSA trajectories exhibited more behavioral problems (e.g., withdrawal, aggression, oppositional defiance), and poorer social responsiveness, and all children with parent-reported developmental delay or ASD exhibited atypical RSA growth trajectories (21, 22).

According to Porges' polyvagal theory (3), healthy ANS function, driven primarily by PNS-mediated vagal regulation of the heart, supports social engagement and social learning. Given that ASD is characterized primarily by social and social-communicative impairments, much prior research has focused on non-syndromic ASD, and ANS dysfunction in individuals with non-syndromic ASD is fairly well established. Most studies have shown that children with ASD exhibit elevated HR and lower HRV at baseline, suggesting attenuated PNS function and subsequent chronic hyperarousal, as well as atypical reactivity to stressors [for thorough reviews, see (19, 23–25). In children with ASD, ANS dysfunction has been linked with poorer social functioning, higher sensory sensitivity, greater anxiety, and poorer emotion recognition (19, 21, 23, 26–30). Furthermore, recent work has suggested that reduced SNS activity differentiates children with ASD with and without anxiety, whereas reduced PNS activity differentiates children with ASD from typically developing children (31). In the only longitudinal study of baseline ANS function in ASD, Sheinkopf and colleagues (32) found that children with ASD exhibited less change in RSA across early childhood, with significantly lower RSA evident as early as 18 months, while HR developed similarly in typically developing (TD) children and children with ASD. However, this study focused on infants at high risk for ASD because of prenatal drug exposure, with only 12 children with ASD in the sample. Thus, it is difficult to generalize these findings or draw conclusions about how ANS function develops across infancy and early childhood in the majority of children with ASD.

It has been hypothesized that ANS dysfunction plays a significant role in anxiety disorders as well (33, 34). For example, in the autonomic inflexibility model, Friedman (33) posits that biological competence is characterized by strong vagal regulation (i.e., high HRV), which consequently permits adaptive physiological and behavioral response to stress or fear followed by rapid physiological recovery. When vagal regulation is reduced or disrupted, as reflected by lower HRV, physiological responding becomes chronically blunted, rigid, and predictable, and physiological recovery is slower, ultimately leading to the emergence and maintenance of anxious symptoms. In support of this theory, lower baseline HRV has been associated with greater anxiety symptoms in children (35), and children with anxiety disorders exhibit lower baseline HRV than those without (36, 37). Several studies have also reported elevated baseline HR in children with social anxiety symptoms, suggesting that chronic hyperarousal plays a role in social anxiety (37–39). To our knowledge, no studies have examined how ANS maturation across early development relates to later anxiety symptoms in either typical development or neurodevelopmental disorders.

ANS dysfunction has also been implicated in fragile X syndrome (FXS), a monogenic disorder cause by a CGG trinucleotide expansion mutation on the *FMR1* gene on the X chromosome. Fragile X syndrome is relatively rare, affecting approximately 1 in 7,000 males and 1 in 11,000 females (40). It is characterized by moderate intellectual disability, behavior problems, and sensory processing difficulties. Children with FXS are also at significantly increased risk for both ASD (~61%) and anxiety (~40–50%) (41–45), both of which are linked with poorer developmental and social outcomes (46–50). Understanding how ANS dysfunction contributes to increased risk for ASD and anxiety in infants with FXS is of critical importance, as such knowledge will enable early identification of high-risk infants and inform the development of targeted prevention and interventions, which may in turn optimize developmental outcomes and quality of life for children with FXS. Furthermore, the elevated risk for these clinical features along with relatively well understood molecular-genetic and neurobiological mechanisms make FXS an ideal model to improve our understanding of gene-behavior associations related to emerging psychopathology in childhood.

For nearly three decades, the “hyperarousal hypothesis” of FXS has argued that ANS dysfunction underpins many symptoms of FXS (51, 52). Most evidence for hyperarousal in FXS comes from cross-sectional studies of older children and adolescents, which have documented elevated HR and attenuated HRV at baseline as well as atypical reactivity to challenges (30, 53–55). However the emergence, trajectory, and consequences of ANS dysfunction in FXS have not been characterized, thus the developmental origins of ANS dysfunction in FXS remain poorly understood. Despite the limited understanding of ANS dysfunction in FXS, the assumption that “hyperarousal” is a primary causal mechanism for many of the phenotypic features in FXS remains fairly widespread. For example, according to an expert consensus document (56) published on the National Fragile X Foundation website (www.fragilex.org), “Individuals with FXS are always susceptible to hyperarousal,” “…hyperarousal…is believed to underlie many of the phenotypical behaviors commonly associated with fragile X syndrome,” and “When problem behaviors occur, it is important to recognize that they may be caused by hyperarousal…” However, surprisingly little research has directly examined the relationship between ANS dysfunction and the FXS behavioral phenotype, particularly using longitudinal methods in early development. Roberts et al. (57) documented elevated HR and lower RSA in the youngest sample to date (8–40 months of age), and found that lower RSA was associated with greater ASD symptoms in children aged 22 months or older, but not in younger children. In the only longitudinal study to date in FXS, Roberts et al. (41) found that shorter initial IBI in infancy, but not change over time, predicted later ASD diagnoses in young children with FXS. Interestingly, neither initial RSA nor change over time in RSA predicted ASD diagnoses. These findings are surprising given that PNS dysfunction, as indexed by HRV and RSA, is thought to support social engagement and social learning (3) and has been consistently linked to non-syndromic ASD (19, 24).

In conclusion, despite the long-standing “hyperarousal hypothesis” in FXS, most previous studies have examined ANS function in FXS cross-sectionally across a limited age range. Furthermore, there has been little emphasis on infancy, developmental change across early childhood, or the developmental consequences of early ANS dysfunction on later psychopathology. The first aim of this longitudinal study was to characterize developmental change in ANS function across infancy and early childhood in FXS and typical development using the cardiac indices of IBI and RSA. Based on previous cross-sectional work in children with FXS, we hypothesized that a profile of hyperarousal, indexed by shorter IBI and lower RSA, emerges early in life increases in severity across early development in FXS. We also hypothesized that early ANS dysfunction has important developmental repercussions, conferring heightened risk for later ASD and anxiety symptoms. Thus, the second aim of the study was to examine the relationship between early ANS function and developmental change in ANS function with later ASD and anxiety symptoms. Based on leading theories of the role of ANS dysfunction in the development of anxiety (33, 34) and ASD symptoms (3, 25), we predicted that shorter IBI and slower change in IBI across development would be related to later anxiety symptoms, while lower RSA and slower change in RSA across development would be related to later ASD symptoms.

## Materials and Methods

### Participants

This study included 152 male children (FXS: *n* = 73; TD: *n* = 79). Baseline heart activity was measured longitudinally between 3 and 83 months of age, resulting in 372 observations (FXS: *n* = 175; TD *n* = 197). Groups were matched on average chronological age, *t*_(370)_ = 0.06, *p* = 0.954, and exhibited similar distributions of observations across age. This age range was selected to maximize the number of longitudinal heart activity data points available per participant in order to address our first research question of characterizing the emergence and trajectory of autonomic dysfunction across infancy and early childhood (i.e., infancy through six years of age). Participant characteristics are detailed in Table 1. Participants were drawn from two sites that collected baseline heart activity data longitudinally across infancy and early childhood. Data for 77 children (FXS: *n* = 36; TD *n* = 41) children were collected at the University of North Carolina at Chapel Hill (P30HD003110; PI: Bailey), and data for 75 children (FXS: *n* = 37; TD *n* = 38) were collected at the University of South Carolina (R01MH107573, R01MH090194; PI: Roberts). Thus, this is a convenience sample that provides the opportunity to analyze the developmental trajectory of ANS function from a large sample of infants and children with a rare genetic disorder. As a result, the present study represents the largest longitudinal sample of males with FXS (*n* = 73) spanning the most inclusive age range across early development (3–83 months) to date. Heart activity collection and processing procedures were identical at both study sites and were designed and overseen by the senior author. Some of the data included in the present study have been included in previous cross-sectional studies (53, 58–60) and a recent longitudinal study that examined various predictors of ASD diagnoses in preschool-aged males and females with FXS (41). The objectives and analyses of the present study are quite different from those of previous studies, and no studies have been published that focus on characterizing ANS function using longitudinal data across this age range or with such a large sample.

**Table 1:** Participant characteristics: research question 1.

	**FXS (*n =* 73)**	**TD (*n =* 79)**
Total number of observations	175	197
Observations per participant, M (SD)	2.40 (1.38)	2.49 (1.89)
Observations per participant, Range	1–6	1–7
Chronological age (months), M (SD)	33.96 (21.85)	34.10 (23.97)
Chronological age (months), Range	3.05–82.99	5.67–83.81

Recruitment procedures and inclusionary criteria were similar across sites. Children with FXS were recruited through local and national organizations that serve children with FXS. Diagnosis of FXS was confirmed through genetic report (i.e., >200 CGG repeats on 5′UTR on *FMR1*). TD children were recruited through advertisements placed in the community and were required to have no family history of ASD, FXS, or related disorders. Typical development was confirmed by direct clinical-behavioral testing through the larger studies, and was defined by a standard score of ≥70 on the Mullen Scales of Early Learning [MSEL; (61)] or Differential Abilities Scale, Second Edition [DAS-II; (62)] and case review via a thorough clinical best estimate review procedure (41, 63). Participants were excluded if they were born at <37 weeks gestation, had uncorrected vision or hearing impairments, or had parents who were not proficient in English.

To investigate our second research question of whether early ANS function or developmental change in ANS function across early development predicted later ASD or anxiety symptoms, we included a subset of children from the larger sample who had at least two heart activity data points and completed clinical-behavioral “outcome” testing at ≥36 months of age (FXS: *n* = 28; TD: *n* = 25). These children were drawn exclusively from the University of South Carolina study, as that study included outcome assessments of ASD and anxiety symptoms using gold-standard diagnostic assessment procedures. The groups did not differ on chronological age at the outcome timepoint, although, as expected, they did differ on developmental level and ASD symptom severity (see Table 2).

**Table 2:** Participant characteristics at outcome timepoint: research question 2.

	**FXS (*n =* 28)**	**TD (*n =* 25)**	***t*_**(51)**_**	***p*-value**
Chronological age (months), M (SD)	63.96 (12.04)	63.22 (12.49)	−0.22	0.828
Chronological age (months), Range	46.76–83.55	35.78–83.81		
Developmental level, M (SD)	51.39 (9.29)	102.08 (13.78)	15.85	<001
Developmental level, Range	30–76	70–130		
ASD Diagnosis, *n* (%)	20 (71.43%)	0 (0%)		
ADOS-2 CSS, M (SD)	6.07 (2.28)	1.84 (1.31)	−8.16	<001
ADOS-2 CSS, Range	1–10	1–6		
PAS total raw score, M (SD)	16.04 (9.03)	12.08 (7.41)	−1.73	0.090
PAS total raw score, Range	0–39	3–25		

### Measures

#### Heart Activity

Heart activity data were collected during a baseline period with a telemetry-based monitor (Alive Technologies, Copyright 2005–2009; CamNtech Ltd., Cambridge, UK). Infants aged 3–30 months sat on a parent's lap or in a highchair and watched a 3-min Baby Einstein video and children aged 31–83 months sat in a chair and watched a 5-min Pixar animated short film. Both videos contained music and sound effects, but no spoken language. Interbeat interval (i.e., IBI, the time in milliseconds between two consecutive heartbeats) was visually inspected and edited to correct arrhythmias, false heart periods, and artifacts by trained research assistants using CardioEdit software (Brain-Body Center, University of Illinois at Chicago). Heart activity data were excluded if >10% of the file was edited. In this sample, an average of 1.09% of IBI values were edited per file. Respiratory sinus arrhythmia (RSA) was extracted from the edited IBI data using CardioBatch software (Brain-Body Center, University of Illinois at Chicago). CardioBatch samples sequential heart periods at 250 ms epochs and then uses a 21-point moving polynomial algorithm to detrend the data (64). The data are bandpass filtered to extract variance associated with the spontaneous breathing frequency for infants aged 3–30 months (0.3–1.3 Hz) or for children aged 31–83 months (0.24–1.04 Hz). The variance is then transformed into its natural logarithm to provide an estimate of RSA. Mean IBI and mean RSA for the entire baseline period were computed in CardioBatch and used as dependent variables in this study.

#### ASD Symptoms

The Autism Diagnostic Observation Schedule, Second Edition [ADOS-2; (65)] was administered by a research-reliable examiner to assess ASD symptom severity at the outcome timepoint. The ADOS-2 is a play-based, semi-structured measure developed for children and adults older than 12 months of age. The overall Calibrated Severity Score (CSS) provided a continuous index of ASD symptom severity. Scores range from 1 to 10, with higher values reflecting more severe ASD symptoms. To investigate inter-rater agreement, 18.9% of ADOS administrations in the present study were coded by a second research-reliable examiner, and an intraclass correlation (ICC) of 0.90 on overall CSS indicated excellent agreement.

#### Anxiety Symptoms

Anxiety symptoms were assessed at the outcome timepoint using the total raw score from the Preschool Anxiety Scale [PAS; (66)]. The PAS is a 28-item parent-report questionnaire for preschool-aged children designed to assess symptoms of anxiety and worry. The PAS demonstrates good construct, predictive, and discriminant validity as well as satisfactory internal consistency (57). Total raw scores range from 0 to 112, with higher scores indicating more severe anxiety symptoms.

#### Developmental Level

Developmental level was assessed at the outcome timepoint for children ≤68 months old (*n* = 33) via the Mullen Scales of Early Learning (MSEL; 64), a developmental measure designed to assess cognitive abilities in infants and children up to 68 months of age. The early learning composite (ELC), a composite score of the Visual Reception, Fine Motor, Expressive Language, and Receptive Language subscales, was used. The Differential Abilities Scale, Second Edition – [DAS-II; (62)] was used to assess developmental level in children >68 months old (*n* = 20). The DAS-II is designed to measure cognitive ability in children between 2 years, 6 months and 17 years, 11 months. Developmental level was estimated via the General Conceptual Ability (GCA) score, which is a composite of the Verbal Reasoning, Nonverbal Reasoning, and Spatial Abilities subscales.

### Procedure

Procedures were approved by the Institutional Review Boards at both sites (University of North Carolina at Chapel Hill: #05-0106, initial date of approval 3/17/2003; University of South Carolina: #00001966, initial date of approval 4/30/2009). Informed consent was obtained from the parents before study enrollment. Assessments occurred at participants' homes or in research laboratories. Baseline ECG was collected at the beginning of the assessment. Parents received monetary compensation for their participation.

### Statistical Analyses

Developmental change in IBI and RSA across early childhood was characterized using separate hierarchical linear models (HLMs) via SAS PROC MIXED in SAS 9.4 (SAS Institute Inc., Cary, NC). A model-building approach was utilized to determine the most parsimonious, best-fitting model (67). The results of all models considered are detailed in the Supplementary Materials. For both IBI and RSA, the best fitting model included the fixed and random effects of age (grand-mean centered at 34.04 months), fixed effects of group and site and the age by group interaction. Importantly, the model that included the age by group by site interaction term was a poorer fit and the interaction was non-significant, indicating that group differences in developmental change in RSA and IBI were similar across both sites.

In our second research question, which investigated whether early ANS function or developmental change in ANS function across early development predicted ASD or anxiety symptoms, the model was recentered at 24 months and slope and intercept values were extracted for each participant. This age was selected to represent a timepoint earlier in development that could be used to predict ASD and anxiety symptoms measured at ≥36 months, when ASD symptoms are considered reliable and stable and anxiety symptoms have begun to emerge. Four separate multivariate general linear models were employed in SPSS 27 (SPSS Inc., Chicago, IL) to examine how IBI intercept, IBI slope, RSA intercept, and RSA slope predicted ASD symptoms (i.e., ADOS-2 CSS score) and anxiety symptoms (i.e., PAS total raw score). Group was entered as a fixed factor and the group by heart activity variable (e.g., group by IBI intercept) was included initially to determine whether heart activity predicted symptoms differently between groups. The interaction term was non-significant in every model, so results from main effects models are reported in text. Results of non-significant interaction models are included in the Supplementary Materials. Developmental level was included as a covariate in these models, as it differed between groups and was correlated with RSA slope, *r* =.29, *p* = 0.026, and ASD symptom severity, *r* = −0.69, *p* < 0.001, and was marginally correlated with RSA intercept, *r* = 0.26, *p* = 0.053.

## Results

### Change in ANS Function Across Early Development

#### IBI

**Figure 1:**
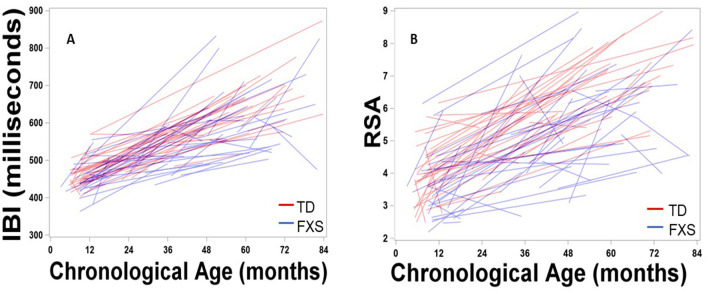
Maturation of **(A)** IBI and **(B)** RSA across age within each participant. Males with FXS are indicated in blue; TD males are indicated in red.

**Figure 2:**
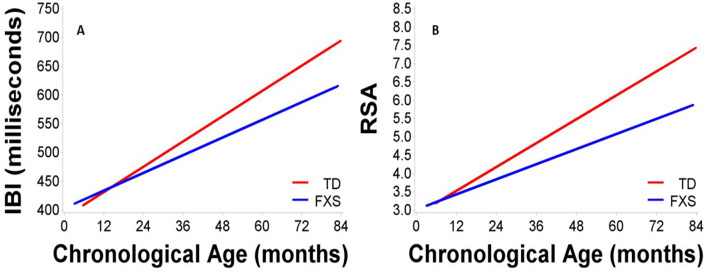
Maturation in **(A)** IBI and **(B)** RSA across age in males with FXS and TD males. Males with FXS are indicated in blue; TD males are indicated in red.

Figure 1 depicts developmental change in IBI and RSA across age within each participant. Significant main effects of group, *F*_(1,132)_ = 8.65, *p* = 0.004, age, *F*_(1,111)_ = 313.11, *p* < .001, and site, *F*_(1,163)_ = 34.77, *p* < 0.001, were observed. A significant group by age interaction was observed, *F*_(1,116)_ = 9.25, *p* = 0.003, with IBI increasing more each month in TD children than in FXS, *b* = 1.10ms (Table 3, Figure 2). The FXS group exhibited significantly shorter IBI by 29 months, *t*_(139)_ = −2.12, *p* = 0.036.

**Table 3:** Estimates for Hierarchical Linear Models of IBI and RSA across early development.

	**IBI**	**RSA**
**Fixed effects**		
Intercept	512.06^***^ (7.32)	4.72^***^ (0.15)
Age	3.66^***^ (0.24)	0.05^***^ (0.00)
Group (FXS)	−21.75^**^ (7.40)	−0.54^***^ (0.16)
Age*Group (FXS)	−1.10^**^ (0.36)	−0.02^***^ (0.01)
Site (USC)	45.88^***^ (7.78)	0.70^***^ (0.16)
**Error variance**		
Intercept	682.16^***^ (218.82)	00.47^***^ (0.10)
Slope	1.68^***^ (0.49)	0.00^‡^ (0.00)
*ICC* (from Model 1)	0.14	0.31

‡ *p < 0.10;*

** *p < 0.01;*

*** *p < 0.001; n = 372; Values represent parameter estimates with standard errors in parentheses; Estimation method = Full; Covariance structure = Variance components*.

#### RSA

Results indicated significant main effects of group, *F*_(1,150)_ = 11.88, *p* < 0.001, age, *F*_(1,109)_ = 268.19, *p* < 0.001, and site, *F*_(1,172)_ = 18.94, *p* < 0.001. A significant group by age interaction was observed, *F*_(1,109)_ = 12.01, *p* < 0.001, suggesting that RSA increased more each month in TD children than in FXS children, *b* = 0.02 (Table 3, Figure 2). The FXS group exhibited significantly lower RSA by 24 months, *t*_(150)_ = −2.10, *p* = 0.037.

### Relationship Between ANS Function at 24 Months and ASD and Anxiety Symptoms at ≥36 Months

#### IBI

**Figure 3:**
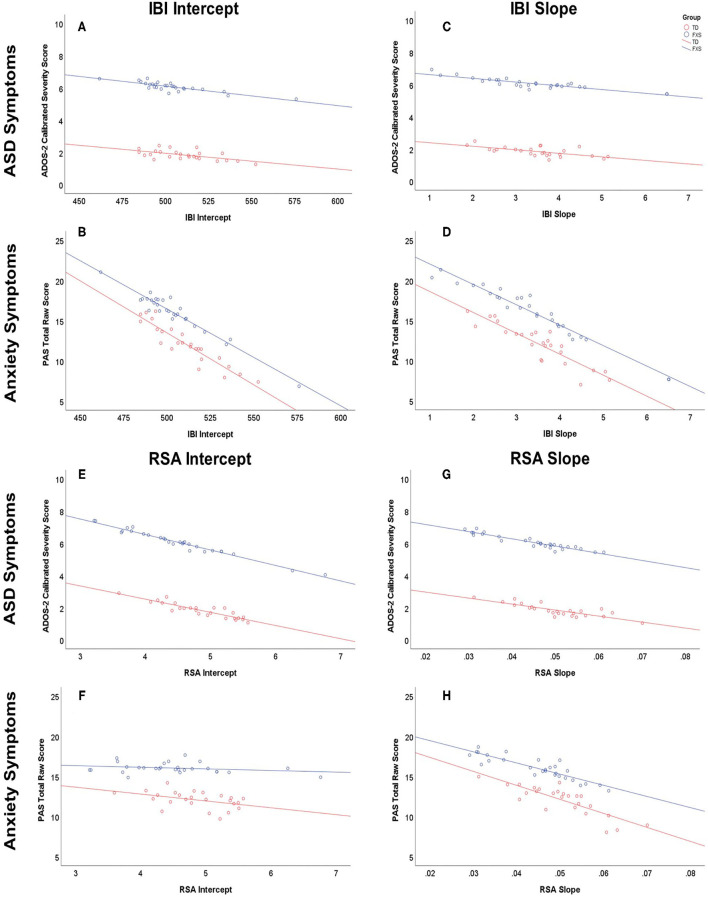
Relationship between IBI and RSA at 24 months of age and ASD and anxiety symptoms at 36 months of age or older (predicted values). Males with FXS are indicated in blue; TD males are indicated in red. IBI intercept predicting ASD and anxiety symptoms is depicted in **(A)** and **(B)**, respectively. IBI slope predicting ASD and anxiety symptoms is depicted in **(C)** and **(D)**, respectively. RSA intercept predicting ASD and anxiety symptoms is depicted in **(E)** and **(F)**, respectively. RSA slope predicting ASD and anxiety symptoms is depicted in **(G)** and **(H)**, respectively.

Full multivariate general linear model results are outlined in Table 4. IBI intercept was a marginally significant predictor in the overall model, *F*_(2,48)_ = 2.78, *p* = 0.072. The main effect of group was significant, *F*_(2,48)_ = 4.28, *p* = 0.019. Tests of between-subjects effects indicated that IBI intercept predicted anxiety symptoms, *F*_(1,49)_ = 4.59, *p* = 0.037, but not ASD symptoms, *F*_(1,49)_ = 0.67, *p* = 0.419 (Figures 3A,B). Group predicted ASD symptoms, *F*_(1,49)_ = 6.58, *p* = 0.013, but not anxiety symptoms, *F*_(1,49)_ = 1.45, *p* = 0.235. Parameter estimates suggested that for every 1ms increase in IBI intercept (i.e., IBI at 24 months), anxiety scores decreased by 0.12 points. The effect of IBI slope emerged as a significant predictor in the model, *F*_(2,48)_ = 4.05, *p* = 0.024, as did group, *F*_(2,48)_ = 4.96, *p* = 0.011. Between-subjects tests indicated that IBI slope predicted anxiety, *F*_(1,49)_ = 6.75, *p* = 0.012, but not ASD symptoms, *F*_(1,49)_ = 0.85, *p* = 0.362 (Figures 3C,D). Group predicted ASD symptoms, *F*_(1,49)_ = 7.04, *p* = 0.011, but not anxiety symptoms, *F*_(1,49)_ = 2.06, *p* = 0.158. Parameter estimates indicated that for every 1ms increase per month in IBI, anxiety scores decreased by 2.60 points.

**Table 4:** Multivariate general linear model results for IBI intercept and IBI slope at 24 months predicting later ASD and anxiety symptoms.

	**IBI Intercept**				**IBI Slope**			
	**λ**	***F*_**(2,48)**_**	***p*-value**	** $\eta_{\text{p}}^{2}$ **	**λ**	***F*_**(2,48)**_**	***p*-value**	** $\eta_{\text{p}}^{2}$ **
Intercept	0.85	4.30	0.019	0.15	0.76	7.75	0.001	0.24
IBI	0.90	2.78	0.072	0.10	0.86	4.05	0.024	0.14
Group	0.85	4.28	0.019	0.15	0.83	4.96	0.011	0.17
Developmental Level	0.98	0.48	0.624	0.02	0.98	0.53	0.592	0.02

*λ = Wilks' lambda; $\eta_{p}^{2}$ = partial eta squared*.

#### RSA

RSA intercept emerged as a marginally significant overall predictor, *F*_(2,48)_ = 3.10, *p* = 0.054 (Table 5). The main effect of group was significant, *F*_(2,48)_ = 4.43, *p* = 0.0174. Tests of between-subjects effects indicated that RSA intercept predicted ASD symptoms, *F*_(1,49)_ = 6.13, *p* = 0.017, but not anxiety symptoms, *F*_(1,49)_ = 0.06, *p* = 0.802. Group predicted ASD symptoms, *F*_(1,49)_ = 6.79, *p* = 0.012, but not anxiety symptoms, *F*_(1,49)_ = 1.66, *p* = 0.203 (Figures 3E,F). Parameter estimates suggested that for every 1-unit increase in RSA at 24 months, ASD severity scores decreased by 0.91 points (Figure 3E). RSA Slope was not a significant predictor in the overall model, *F*_(2,48)_ = 1.86, *p* = 0.166 (Figures 3G,H), while group was, *F*_(2,48)_ = 4.23, *p* = 0.020. Group predicted ASD symptoms, *F*_(1,49)_ = 6.45, *p* = 0.014, but not anxiety symptoms, *F*_(1,49)_ = 1.48, *p* = 0.229.

**Table 5:** Multivariate general linear model results for RSA intercept and RSA slope at 24 months predicting later ASD and anxiety symptoms.

	**RSA intercept**				**RSA slope**			
	**λ**	***F*_**(2,48)**_**	***p*-value**	** $\eta_{\text{p}}^{2}$ **	**λ**	***F*_**(2,48)**_**	***p*-value**	** $\eta_{\text{p}}^{2}$ **
Intercept	0.74	8.57	0.001	0.26	0.77	7.09	0.002	0.23
RSA	0.89	3.10	0.054	0.12	0.93	1.86	0.166	0.07
Group	0.84	4.43	0.017	0.16	0.85	4.23	0.020	0.15
Developmental Level	0.98	0.42	0.658	0.02	0.98	0.46	0.635	0.02

*λ = Wilks' lambda; $\eta_{\text{p}}^{2}$ = partial eta squared*.

## Discussion

Findings from the present study indicate that physiological hyperarousal is present by 24 months of age in males with FXS and that trajectories of hyperarousal across infancy to early childhood differentially predict ASD and anxiety symptoms. This study is the largest longitudinal study to document the developmental emergence of hyperarousal in very young children with FXS, and it provides important evidence in support of the “hyperarousal hypothesis” in FXS (51). While previous cross-sectional studies (30, 53–55, 57, 68) were critical in establishing evidence of atypical ANS function in FXS, until now the lack of dense longitudinal sampling early in development has precluded conclusions about the developmental emergence of the hyperarousal profile so often considered to be a primary source of impairments in FXS [e.g., (56)]. The emergence of hyperarousal early in development, as observed in the present study, has important long-term developmental repercussions as the first years of life represent a developmental period where multiple critical social, emotional, regulatory, and communication skills are rapidly developing alongside extensive brain maturation in a dynamic inter-dependent manner [e.g., (69)].

We were somewhat surprised that the FXS group was not statistically distinguishable from the TD group on cardiac indices earlier in infancy, given that other FXS-related developmental differences are evident earlier in development. While ANS dysfunction may originate earlier in infancy or even in prenatal development, it is possible that our relatively small number of datapoints under 12 months of age (*n* = 26), with the median age of first datapoint of 21 months, limited our statistical ability to identify differences in ANS function in infancy. It is worth noting that marginally significant group differences began to emerge at 27 months for IBI and 21 months for RSA, suggesting that perhaps with additional longitudinal data in infancy, we may have been able to detect group differences earlier in development. However, with more than half of participants (*n* = 82, 53.95%) entering the study before their second birthday (FXS: 53.42%; TD: 54.47%), our sample still represents the largest study to date of ANS function in early development in FXS. Future studies should consider denser longitudinal sampling throughout the first year of life to document the earliest potential onset and trajectory of ANS dysfunction in FXS. Understanding the timing and nature of ANS dysfunction as early as possible in infancy will provide critical opportunities for early identification, prevention, and intervention.

The relationship between early developmental trajectories of hyperarousal to increased psychiatric symptom severity later in development support the critical role that ANS function has on the FXS phenotype, which is characterized by high rates of both anxiety and ASD. Specifically, shorter IBI at 24 months and slower change in IBI across development predicted higher anxiety symptoms, but not ASD symptoms. The relationship between early hyperarousal and later anxiety symptoms is in line with the theory of “autonomic inflexibility” (33, 34) and empirical work that has documented the relationship between elevated baseline arousal and anxiety in neurotypical children (37–39). Generally, these studies suggest that anxiety is characterized by heightened arousal and reduced parasympathetic activity at baseline as well as blunted reactivity to and delayed recovery from stress. While our study did not investigate autonomic reactivity or recovery, previous cross-sectional studies of autonomic reactivity to social stress in FXS have yielded conflicting results that are suggestive of developmental shifts in both baseline activity and autonomic reactivity in FXS across infancy and early childhood. For example, 12-month-old infants with FXS have been shown to exhibit normative baseline RSA but reduced RSA reactivity to a stranger approach (60), whereas young children with FXS exhibit shorter IBI and lower RSA at baseline but typical patterns of reactivity and recovery when approached by a stranger (58). Interestingly, shorter IBI was associated with more behavioral indicators of fear in younger children (<29 months), while longer IBI was associated with more behavioral indicators of fear in older children (>51 months). These findings, taken together with the findings of the present study, highlight the importance of longitudinal work in elucidating the developmental emergence and developmental trajectory of autonomic dysfunction in FXS. Furthermore, while our findings suggest a relationship between developmental change in baseline hyperarousal and later anxiety symptoms, much remains to be understood about the role that autonomic reactivity to and recovery from stress plays in the development of anxiety symptoms in children with FXS.

In the present study, lower RSA at 24 months predicted greater ASD symptoms, but not anxiety symptoms. Our findings of a relationship between lower baseline RSA and higher ASD symptoms parallel converging evidence of poor parasympathetic regulation in children with non-syndromic ASD [for thorough reviews, see (19, 23–25)]. These findings, in combination with our own that document a relationship between early RSA, but not early IBI, and later ASD symptoms, suggest that parasympathetic activity may play an important role in ASD, whereas overall arousal may be less relevant. This is in line with leading theories (e.g., polyvagal theory) that argue that PNS-mediated regulation of the vagal nerve supports social engagement, social learning, and social-emotional development (3). Our findings also correspond with recent research that suggests that lower parasympathetic activity can differentiate children with ASD from TD controls but does not differentiate anxious and non-anxious children with ASD (31). Previous studies of FXS have also suggested that parasympathetic activity may also predict ASD symptoms, but the findings have been nuanced. For example, IBI has been shown to predict later ASD symptoms in a non-linear fashion across age, with greater ASD severity in childhood being associated with longer IBI in infancy but shorter IBI in childhood (57). In the only longitudinal study of ANS function in young children with FXS to date, Roberts and colleagues (41) found that shorter initial IBI, but not developmental change in IBI, predicted later ASD diagnoses in young children with FXS. Interestingly, neither initial RSA nor RSA maturation predicted ASD diagnoses. It is important to note that some data from these previous studies are included in the convenience sample used in the present study, but there are several important methodological differences that may explain why our findings differed. For example, Roberts and colleagues (41) examined categorical diagnostic outcomes while we used a continuous measure of ASD symptom severity which may have provided more variability and statistical power. Both of these previous studies also included females in their study, while the present study was limited to males, who tend to be more severely impaired and have higher ASD symptom severity (70).

Surprisingly, developmental change in RSA did not predict ASD symptoms later in childhood, despite findings of developmental change in RSA being linked with later ASD symptoms in previous studies of low-risk and high-risk infants (21, 22, 32). For example, slower developmental change in RSA has been linked with later ASD diagnoses in infants with prenatal drug exposure (32). Slower or plateauing developmental change in RSA has also been linked with behaviors associated with ASD, such as behavioral problems (e.g., withdrawal, aggression, oppositional defiance) and poorer social responsiveness (21, 22). The findings of the present study suggest that it is RSA in early development, not growth or change over time, that has long-term impact on ASD symptoms.

### Limitations and Future Directions

This study is the largest longitudinal study of ANS function in FXS to date, and it is the first to examine developmental emergence and change in ANS function across infancy and early childhood in relation to symptoms of anxiety of ASD, which are the most common psychiatric comorbidities of FXS. Despite our important findings, certain limitations should be considered. First, the nature of our convenience sample compiled from multiple larger studies was both a strength, in that it enabled us to examine ANS function in a large sample of infants and children with a rare genetic disorder, and a weakness, in that some measures (e.g., outcome measures) were available in only a subset of participants. Additionally, we did not have detailed molecular-genetic data (e.g., FMRP, methylation) on most of our participants, so we were unable to investigate the relationship between molecular-genetic variation and physiological hyperarousal. These analyses would provide valuable information about the pathophysiological mechanisms underlying atypical development in FXS and should be considered in future studies.

Examining early development over a large age range also posed certain methodological challenges, including discontinuity of measures across development. For example, in our study, the Mullen Scales of Early Learning (61) was used as a measure of developmental level through early childhood, but it is only appropriate until 68 months of age, at which point we used the Differential Abilities Scale–Second Edition [DAS-II; (62)]. However, because identical procedures were used in both FXS and TD, and developmental level served as a covariate at the outcome timepoint and not a primary variable of interest, we believe this is a minor limitation. Finally, this study was limited to males, and the exclusion of females with FXS restricts the generalizability of findings to all children with FXS. Because of their unique clinical profiles and the fact that FXS is less common in females (40), females have often been overlooked in previous studies (71). Work focused on characterizing and understanding ANS function in females with FXS is desperately needed, and we intend to address that critical gap in the future.

### Summary and Implications

Findings indicate that ANS dysfunction and hyperarousal emerge by 24 months of age in males with FXS. Importantly, hyperarousal across early childhood predicts elevated symptom severity for both anxiety and ASD, which is in line with the “hyperarousal hypothesis” of FXS and clearly documented negative impact of hyperarousal on multiple domains of function in non-syndromic populations. The findings of the present study have several important clinical implications. For example, diagnostic efforts should account for the potential impact of hyperarousal on the process and outcome of diagnostic differentiation in children with FXS. Clinicians should consider the role that hyperarousal may be playing in behaviors observed during diagnostic assessments and those reported by parents. Measurement of hyperarousal during the diagnostic process may provide complementary insight into risk for anxiety or ASD symptoms. Additionally, treatment efforts should consider that physiological hyperarousal might represent a modifiable factor that could increase behavioral intervention efficacy. For example, learning to recognize the signs of hyperarousal, directly monitoring arousal in real time, and incorporating calming activities into treatment as needed might be effective approaches to maximizing treatment benefit.

Given that ANS dysfunction, and in particular, attenuated PNS function, has been shown to impact social-communicative development specifically (3), hyperarousal in infancy and early childhood may have detrimental long-term impacts on brain development and social-communicative functioning. However, research on non-syndromic ASD has clearly documented that this developmental period is a prime period for maximum impact of targeted early intervention (72, 73). Furthermore, ANS dysfunction may be an especially promising target for early intervention in FXS, as several treatment methods have been shown to be effective in improving ANS dysfunction and ANS-related symptoms in other disorders. For example, non-invasive transcutaneous vagal nerve stimulation has been shown to be effective in reducing hyperarousal in veterans with posttraumatic stress disorder and traumatic brain injury (74). It is also effective in reducing severity of drug-resistant epilepsy (75) and major depressive disorder (76, 77), and it has been proposed as a potential treatment in non-syndromic ASD (78). Additionally, HRV biofeedback has been shown to be effective in increasing HRV and decreasing symptoms of depression, anxiety, and insomnia in patients with major depressive disorder (79–82), though the cognitive demands of a biofeedback protocol may be difficult for some children with FXS. The findings of the present study suggest that hyperarousal is present very early in development in FXS and has detrimental long-term consequences. Thus, future studies should investigate the efficacy of treatment approaches that directly target ANS dysfunction in FXS.

## Data Availability Statement

The raw data supporting the conclusions of this article will be made available by the authors, without undue reservation.

## Ethics Statement

The studies involving human participants were reviewed and approved by University of South Carolina Institutional Review Board and University of North Carolina at Chapel Hill Institutional Review Board. Written informed consent to participate in this study was provided by the participants' legal guardian/next of kin.

## Author Contributions

AH conceived of study questions, analyzed the data, compiled results, and created figures. AH drafted the manuscript with substantive contributions from EH, KS, CB, and KB. KS oversaw processing of heart activity data. JK and JR aided in interpretation of results and refinement of the manuscript. JR obtained study funding. All authors discussed the results and commented on the manuscript.

## Funding

This work was supported by the National Institute of Child and Human Development (P30HD003110; PI: Bailey) and the National Institute of Mental Health (R01MH107573, R01MH090194; PI: Roberts). This publication was made possible in part by Grant Number T32GM081740 from NIH-NIGMS. Its contents are solely the responsibility of the authors and do not necessarily represent the official views of the NIGMS or NIH. The funders had no role in the study design, data analysis, data interpretation, or writing of this article.

## Conflict of Interest

The authors declare that the research was conducted in the absence of any commercial or financial relationships that could be construed as a potential conflict of interest.

## Publisher's Note

All claims expressed in this article are solely those of the authors and do not necessarily represent those of their affiliated organizations, or those of the publisher, the editors and the reviewers. Any product that may be evaluated in this article, or claim that may be made by its manufacturer, is not guaranteed or endorsed by the publisher.

## Abbreviations

ASD: Autism spectrum disorder
ANS: Autonomic nervous system
FXS: Fragile X syndrome
HR: Heart rate
HRV: Heart rate variability
IBI: Interbeat interval
PNS: Parasympathetic nervous system
RSA: Respiratory sinus arrhythmia
SNS: Sympathetic nervous system.
