# Supplementary material for: Trajectories of Heart Activity Across Infancy to Early Childhood Differentially Predict Autism and Anxiety Symptoms in Fragile X Syndrome
Source: Front Psychiatry. 2021 Oct 6;12:727559. doi: 10.3389/fpsyt.2021.727559 (PMC8526850; doi:10.3389/fpsyt.2021.727559)
Supplement: Supplementary file 1 [file Table_1.DOCX]

|  | Model 1 | Model 2 | Model 3 | Model 4 | Model 5 (Reported in Results) | Model 6 |
| --- | --- | --- | --- | --- | --- | --- |
| *Fixed Effects* |  |  |  |  |  |  |
| Intercept | 532.21*** (5.72) | 528.18*** (4.32) | 542.15*** (5.87) | 515.54*** (7.40) | 512.06*** (7.32) | 520.85*** (9.23) |
| Age | -- | 3.25*** (0.19) | 3.23*** (0.18) | 3.19*** (0.18) | 3.66*** (0.23) | 3.65*** (0.40) |
| Group (FXS) | -- | -- | -27.98** (8.38) | -22.08** (7.61) | -21.75** (7.40) | -41.45** (12.40) |
| Site (USC) | -- | -- | -- | 41.05*** (7.79) | 45.88*** (7.78) | 32.58** (11.13) |
| Age*Group (FXS) | -- | -- | -- | -- | -1.10** (0.36) | -1.76** (0.61) |
| Age*Site (USC) | -- | -- | -- | -- | -- | -0.08 (0.49) |
| Group (FXS)*Site (USC) | -- | -- | -- | -- | -- | 28.94^ǂ^ (15.60) |
| Age*Group (FXS)*Site (USC) | -- | -- | -- | -- | -- | 0.97 (0.75) |
| *Error Variance* |  |  |  |  |  |  |
| Intercept | 1261.15* (733.70) | 1326.03*** (327.58) | 1194.99*** (304.05) | 763.72*** (233.07) | 682.16*** (218.82) | 663.05*** (211.16) |
| Age (Slope) | -- | 1.86*** (0.55) | 1.74*** (0.53) | 1.99*** (0.54) | 1.68*** (0.49) | 1.65*** (0.47) |
| *Model Fit* |  |  |  |  |  |  |
| -2LL | 4450.3 | 4104.8 | 4093.9 | 4069.5 | 4060.8 | 4055.3 |
| AIC | 4456.3 | 4114.8 | 4105.9 | 4083.5 | 4076.8 | 4077.3 |
| BIC | 4465.3 | 4129.9 | 4124.1 | 4104.7 | 4101.0 | 4110.6 |
| *ICC* (Model 1 only) | .14 |  |  |  |  |  |
| Notes: *n*=372; ^ǂ^*p*<.10; ***p*<.01; ****p*<.001; -2LL= -2 log likelihood; AIC=Akaike’s Information Criterion; BIC=Bayesian Information Criterion; Values represent parameter estimates with standard errors in parentheses; Estimation method=Full maximum likelihood; Covariance structure=Variance components. | | | | | | |

Supplemental Table 1. Results from all hierarchical linear models considered during the model-building process for IBI analyses.

Supplemental Table 2. Results from all hieararchical linear models considered during the model-building approach for RSA analyses.

|  | Model 1 | Model 2 | Model 3 | Model 4 | Model 5 (Reported in Results) | Model 6 |
| --- | --- | --- | --- | --- | --- | --- |
| *Fixed Effects* |  |  |  |  |  |  |
| Intercept | 4.94*** (0.11) | 4.85*** (0.09) | 5.15*** (0.12) | 4.25*** (0.14) | 4.72*** (0.15) | 4.72*** (0.18) |
| Age | -- | 0.05*** (0.00) | 0.05*** (0.00) | 0.05*** (0.00) | 0.05*** (0.00) | 0.06*** (0.01) |
| Group (FXS) | -- | -- | -0.60*** (0.17) | -0.56*** (0.16) | -0.54*** (0.16) | -0.47^ǂ^ (0.25) |
| Site (USC) | -- | -- | -- | 0.58*** (0.16) | 0.70*** (0.16) | 0.69** (0.23) |
| Age*Group (FXS) | -- | -- | -- | -- | --0.02*** (0.00) | -0.01 (0.01) |
| Age*Site (USC) | -- | -- | -- | -- | -- | -0.00 (0.01) |
| Group (FXS)*Site (USC) | -- | -- | -- | -- | -- | 0.00 (0.32) |
| Age*Group (FXS)*Site (USC) | -- | -- | -- | -- | -- | -0.02 (0.01) |
| *Error Variance* |  |  |  |  |  |  |
| Intercept | 0.81*** (0.21) | 0.71*** (0.13) | 0.63*** (0.12) | 0.52*** (0.11) | 0.47*** (0.10) | 0.44*** (0.10) |
| Age (Slope) | -- | 0.00^ǂ^ (0.00) | 0.00^ǂ^ (0.00) | 0.00^ǂ^ (0.00) | 0.00^ǂ^ (0.00) | 0.00 (0.00) |
| *Model Fit* |  |  |  |  |  |  |
| -2LL | 1382.8 | 1144.5 | 1132.4 | 1120.5 | 1109.1 | 1104.7 |
| AIC | 1388.8 | 1154.4 | 1144.4 | 1134.5 | 1125.1 | 1126.7 |
| BIC | 1397.9 | 1169.7 | 1162.5 | 1155.7 | 1149.3 | 1160.0 |
| *ICC* (Model 1 only) | .31 |  |  |  |  |  |
| Notes: *n*=372; ^ǂ^*p*<.10; ***p*<.01; ****p*<.001; -2LL= -2 log likelihood; AIC=Akaike’s Information Criterion; BIC=Bayesian Information Criterion; Values represent parameter estimates with standard errors in parentheses; Estimation method=Full maximum likelihood; Covariance structure=Variance components. | | | | | | |

Supplemental Table 3. Multivariate general linear model results for IBI intercept and IBI slope predicting ASD and anxiety symptoms with interactions.

|  | IBI Intercept | | | | IBI Slope | | | |
| --- | --- | --- | --- | --- | --- | --- | --- | --- |
|  | *λ* | *F*(2,47) | *p* | *η*_p_^2^ | *λ* | *F*(2,47) | *p* | *η*_p_^2^ |
| Intercept | .84 | 4.52* | .016 | .16 | .74 | 8.39** | .001 | .26 |
| IBI | .88 | 3.13^ǂ^ | .053 | .12 | .84 | 4.55* | .016 | .16 |
| Group | .94 | 1.52 | .230 | .06 | .98 | 0.40 | .671 | .02 |
| IQ | .88 | 0.33 | .723 | .01 | .98 | 0.52 | .601 | .02 |
| Group*IBI | .94 | 1.44 | .247 | .06 | .97 | 0.63 | .540 | .03 |

Notes: ǂ*p*<.10; **p*<.05; λ=Wilks’ lambda; *η*_p_^2^=partial eta squared

Supplemental Table 4. Multivariate general linear model results for RSA intercept and RSA slope predicting ASD and anxiety symptoms with interactions.

|  | RSA Intercept | | | | RSA Slope | | | |
| --- | --- | --- | --- | --- | --- | --- | --- | --- |
|  | *λ* | *F*(2,47) | *p* | *η*_p_^2^ | *λ* | *F*(2,47) | *p* | *η*_p_^2^ |
| Intercept | .75 | 7.82** | .001 | .25 | .78 | 6.57** | .003 | .22 |
| RSA | .88 | 3.13^ǂ^ | .053 | .12 | .93 | 1.83 | .172 | .07 |
| Group | .96 | 0.95 | .396 | .04 | .98 | 0.53 | .593 | .02 |
| IQ | .99 | 0.24 | .785 | .01 | .98 | 0.41 | .663 | .02 |
| Group*RSA | .94 | 1.47 | .241 | .06 | .99 | 0.01 | .986 | .00 |

Notes: ǂ*p*<.10; **p*<.05; λ=Wilks’ lambda; *η*_p_^2^=partial eta squared
